# Standardised bioassays reveal that mosquitoes learn to avoid compounds used in chemical vector control after a single sub-lethal exposure

**DOI:** 10.1038/s41598-022-05754-2

**Published:** 2022-02-17

**Authors:** Seynabou Sougoufara, Hanna Yorkston-Dives, Nurul Masyirah Aklee, Adanan Che Rus, Jaal Zairi, Frederic Tripet

**Affiliations:** 1 Centre for Applied Entomology and Parasitology, School of Life Sciences, Keele University, Stafforshire, United Kingdom; 2 Vector Control Research Unit, School of Biological Sciences, Universiti Sains Malaysia, George Town, Malaysia

**Keywords:** Ecology, Chemical biology

## Abstract

Vector-borne diseases are worldwide public health issues. Despite research focused on vectorial capacity determinants in pathogen transmitting mosquitoes, their behavioural plasticity remains poorly understood. Memory and associative learning have been linked to behavioural changes in several insect species, but their relevance in behavioural responses to pesticide vector control has been largely overlooked. In this study, female *Aedes aegypti* and *Culex quinquefasciastus* were exposed to sub-lethal doses of 5 pesticide compounds using modified World Health Organization (WHO) tube bioassays. Conditioned females, subsequently exposed to the same pesticides in WHO tunnel assays, exhibited behavioural avoidance by forgoing blood-feeding to ensure survival. Standardized resting site choice tests showed that pre-exposed females avoided the pesticides smell and choose to rest in a pesticide-free compartment. These results showed that, following a single exposure, mosquitoes can associate the olfactory stimulus of pesticides with their detrimental effects and subsequently avoid pesticide contact. Findings highlight the importance of mosquito cognition as determinants of pesticide resistance in mosquito populations targeted by chemical control.

## Introduction

Insects use a range of senses such as sight, smell, taste and hearing to navigate their environment. Under challenging variable conditions, the sensory and cognitive abilities of insects enable them to modulate their behaviour to ensure their survival and reproduction^1^. Learning plays a key role in behavioural plasticity by allowing sensory stimuli to become associated with positive or negative experiences, thereby enabling novel adaptive responses^2,3^. Different insect studies have shown that traits such as foraging, feeding, oviposition and mating all involved associative learning^4–9^.

Understanding learning and memory is important for insects that transmit human diseases, because behaviour such as host finding and blood-feeding have critical implications for their vectorial capacity and thus, disease transmission^10,11^. Vector-borne diseases represent 17% of the global burden of communicable diseases and 80% of the world’s population is at risk from one or more diseases transmitted to humans by a vector^12^. Among them, mosquito-transmitted diseases such as malaria, chikungunya, dengue, yellow fever, Zika and West Nile fever cause millions of cases every year and tropical and subtropical regions are particularly affected^13^. The most important mosquito vectors are *Anopheles, Aedes* and *Culex* species that feed preferentially on humans inside and around their dwellings. In such setting, chemical vector control measures in the form of insecticide treated bed nets (ITNs), indoor residual spraying (IRS) and outdoor space spraying have proved the most effective in reducing mosquito populations and disease transmission^14^.

Unfortunately, decades of reliance on a limited number of pesticide classes with low toxicity to mammals and humans have resulted in mosquito resistance, leaving fewer options for effective control^15^. To remain effective, chemical vector control programs need to closely monitor evolution of pesticide resistance in mosquito populations^15^.

Mosquito resistance to pesticides is typically described as resulting from several non-exclusive mechanisms. Target site insensitivity results from mutations in genes encoding the protein binding sites on which the insecticides act (Kdr, Ace. 1R and Rdl)^16–19^. Metabolic resistance results from overexpression of detoxifying enzymes such as P450 monooxygenases, esterases, and glutathione S transferases (GST) which inactivate insecticides by metabolization or sequestration^20–22^. These two mechanisms in mosquito vectors have been widely described^22^. Additional resistance mechanism can occur through remodeling of the mosquito cuticle by changes in cuticular compounds or thickening leading to decreased insecticide penetration and transport in mosquitoes^23–25^. Behavioural avoidance or 'deterrence' is another mechanism of resistance described as the innate ability of mosquitoes to decrease insecticide exposure by moving away from treated areas^26,27^. This response to insecticides is referred to as 'irritancy', and is elicited following physical contact with a pesticide treated surfaces whereas 'repellency' occurs without contact^28,29^.

Mosquito learning could act as an additional crucial behavioural resistance mechanism that can impact the efficacy of pesticide intervention – this has been suggested by different authors^30–32^. That mosquitoes can learn to associate an olfactory or visual conditioning stimulus (CS) with a positive experience or reinforcing stimulus (RS) has now been demonstrated through laboratory-based individual conditioning studies^31^. For example, males and females of the southern house mosquito *Culex quinquefasciatus* were able to associate an odour not normally encountered in nature (synthetic strawberry or vanilla extracts) with an unconditioned reinforcing stimulus consisting of either a sugar (males and females) or blood (females)^33^. Differential bulk conditioning studies in the malaria mosquito, *Anopheles gambiae* sensu lato showed that these mosquitoes could associate various cues (CS) including visual patterns, and attractive or repellent odours, such as cheese and citronella, with the reward of a bloodmeal (RS), and remember this association for up to 3 days^30^. In the yellow fever mosquito, *Aedes aegypti*, females were shown to temporarily associate colours and odours with the averse stimulus of electroshocks^34^. In other studies, *Ae. aegypti* females positively associated different host odour components with a blood-reinforced thermal stimulus^35^. Crucially, these studies also demonstrated that pre-exposure to the contact repellent DEET resulted in decreased repellence through learning^35^.

Despite accumulating evidence that mosquito learning might play an important part in behavioural avoidance to pesticide, thereby decreasing the efficacy of commonly deployed pesticide intervention, its importance as not yet been formally examined through the standard assays recommended for monitoring pesticide deterrence and resistance^36,37^. In this study, we combined modified WHO assays with WHO tunnel assays and a resting site assay to pre-expose and condition *Ae. aegypti* and *Cx. quinquefasciatus* females to all major pesticide classes used for vector control and then assess the relative importance of deterrence and associative learning responses. The results highlight the importance of learned avoidance to pesticides and are key to our understanding of pesticide avoidance in mosquito populations increasingly resistant to chemical control interventions.

## Results

### Tests of associative learning - WHO tunnel tests

#### Blood-feeding rates

**Figure 1:**
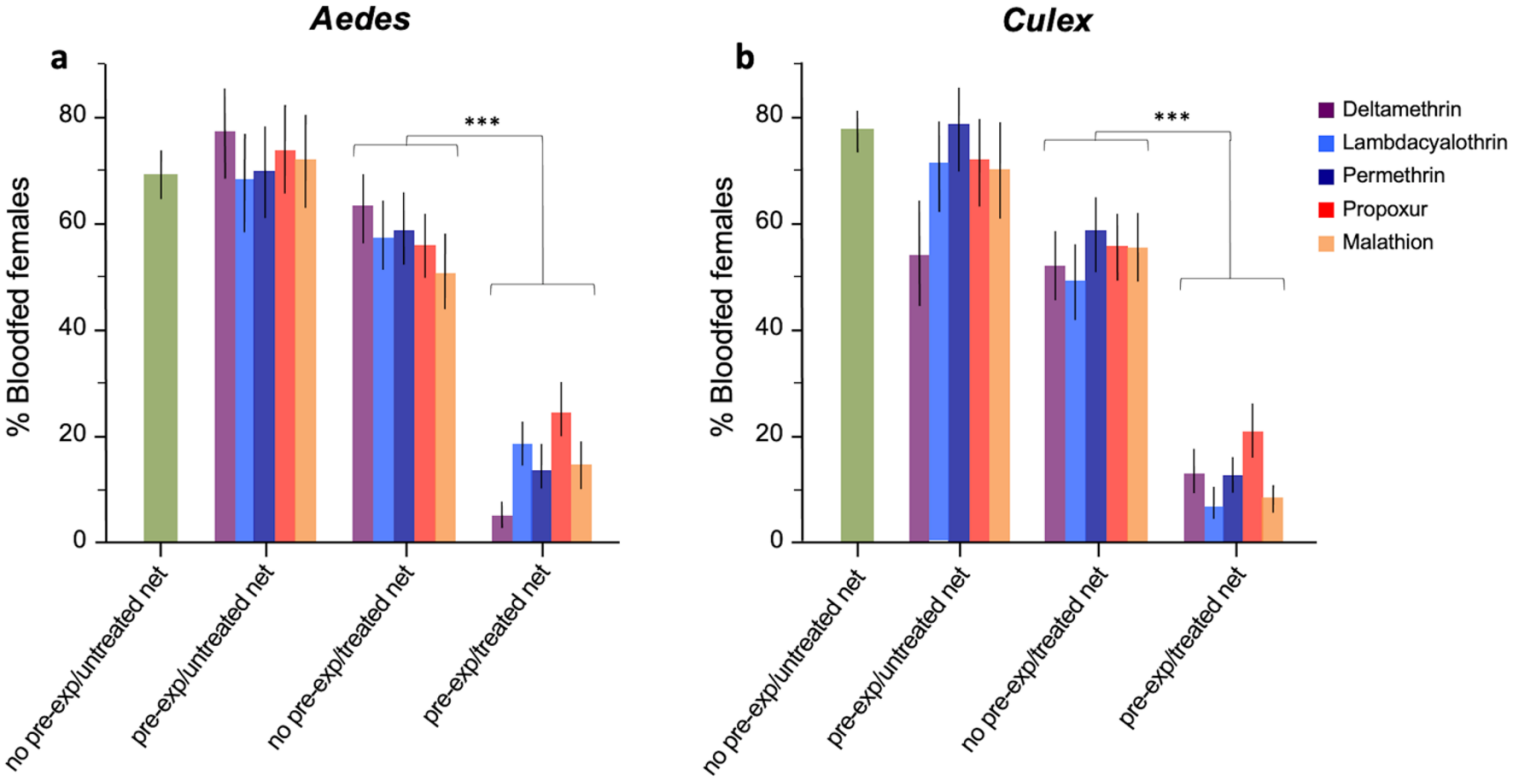
Proportion of blood feeding in (**a**) unconditioned and conditioned *Aedes aegypti* and (**b**) *Culex quinquefasciatus* females exposed to untreated and treated bed nets in the WHO tunnels test. Error bars represent 95% confidence intervals. *P* values are *P* < 0.001 ***.

In *Ae. aegyp*ti 69.1% and 77.2% of *Cx. quinquefasciatus* females that were non-pre-exposed to pesticides fed successfully on the host when faced with an untreated net (Fig. 1a, b). Overall, these percentages did not differ significantly from the feeding rate of pre-exposed *Ae. aegypti* (mean = 72.6% range 68.7–77.2) and *Cx. Quinquefasciatus* (mean = 69.4% range 54.6–78.6) after navigating thorough an untreated net (Likelihood Odds ratio: *P* > 0.05 in all cases), with the exception of deltamethrin in *Cx. quinquefasciatus* (Likelihood Odds ratio: LR = 1.88, *P* = 0.025) (Fig. 1a, b)*.*

Importantly, across all compounds a much lower proportion of pre-exposed female *Aedes aegypti* blood-fed on the host (mean = 15.4% range 5.2–24.9) when faced with a treated net, compared to unconditioned females (mean = 57.7% range: 51.2–63.4) (Fig. 1a, Table 1). The decrease in feeding rate in this treatment group was significant for all pesticides compounds (Likelihood Odds ratio: *P* < 0.001 in all comparisons) and was the highest in Deltamethrin 0.05% and lowest in Propoxur 0.1% (Fig. 1a, Table 1).

**Table 1:** Logistic regressions testing the effects of the 4 treatment groups and replicates on the proportion of females that bloodfed, survived and were found in the host section of the tunnel assay.

Dependent variables	Species	Factors	Deltamethrin		Lambdacyalothrin		Permethrin		Propoxur		Malathion	
			LR^†^	*P* value	LR	*P* value	LR	*P* value	LR	*P* value	LR	*P* value
Bloodfed %	*Ae. aegypti*	Treatment	385.5	< 0.001	242.3	< 0.001	309.0	< 0.001	193	< 0.001	302.7	< 0.001
		Replicate^§^	49.3	< 0.001	88.01	< 0.001	69.8	< 0.001	76.5	< 0.001	72.3	< 0.001
	*Cx. quinquefasciatus*	Treatment	320.1	< 0.001	55.1	< 0.001	405.5	< 0.001	282.1	< 0.001	468.5	< 0.001
		Replicate	67.5	< 0.001	151.7	< 0.001	31.9	< 0.001	27.9	0.0005	25.4	0.001
Host side %	*Ae. aegypti*	Treatment	303.7	< 0.001	168.2	< 0.001	256.8	< 0.001	152.2	< 0.001	213.8	< 0.001
		Replicate	92.5	< 0.001	122.5	< 0.001	163.5	< 0.001	133.7	< 0.001	107.8	< 0.001
	*Cx. quinquefasciatus*	Treatment	376.1	< 0.001	379.3	< 0.001	427.8	< 0.001	300.5	< 0.001	504.1	< 0.001
		Replicate	36.9	< 0.001	41.8	< 0.001	40.7	< 0.001	35.2	< 0.001	21.1	0.007
Survival %	*Ae. aegypti*	Treatment	775.5	< 0.001	299.6	< 0.001	264.6	< 0.001	255	< 0.001	288.2	< 0.001
		Replicate	23.7	0.001	585.3	< 0.001	620.8	< 0.001	600.1	< 0.001	636.5	< 0.001
	*Cx. quinquefasciatus*	Treatment	553.5	< 0.001	219.6	< 0.001	285.1	< 0.001	363.1	< 0.001	369.8	< 0.001
		Replicate	52.2	< 0.001	382.7	< 0.001	348.5	< 0.001	349.1	< 0.001	350.2	< 0.001

All dependent variables were treated as binomial variable. The Chi-square of Likelihood Ratio effect tests (LR) are presented with respective *P* values.

^§^Replicate effects were nested within treatments.

Similar results were observed in *Cx. quinquefasciatus* where the mean values of engorged pre-exposed females, prior exposure to pesticides was much lower (mean 12.1% range 6.9—21.1) compared to the non-pre-exposed females (mean = 54.4% range 49.5–58.3) (Fig. 1b). Logistic regressions showed that the differences in feeding rate were significant for all insecticides tested (Likelihood Odds ratio: *P* < 0.002 in all cases, Table 1) and were largest when females were exposed to Malathion and lowest with Propoxur (Table 1, Fig. 1b).

#### Host side

**Figure 2:**
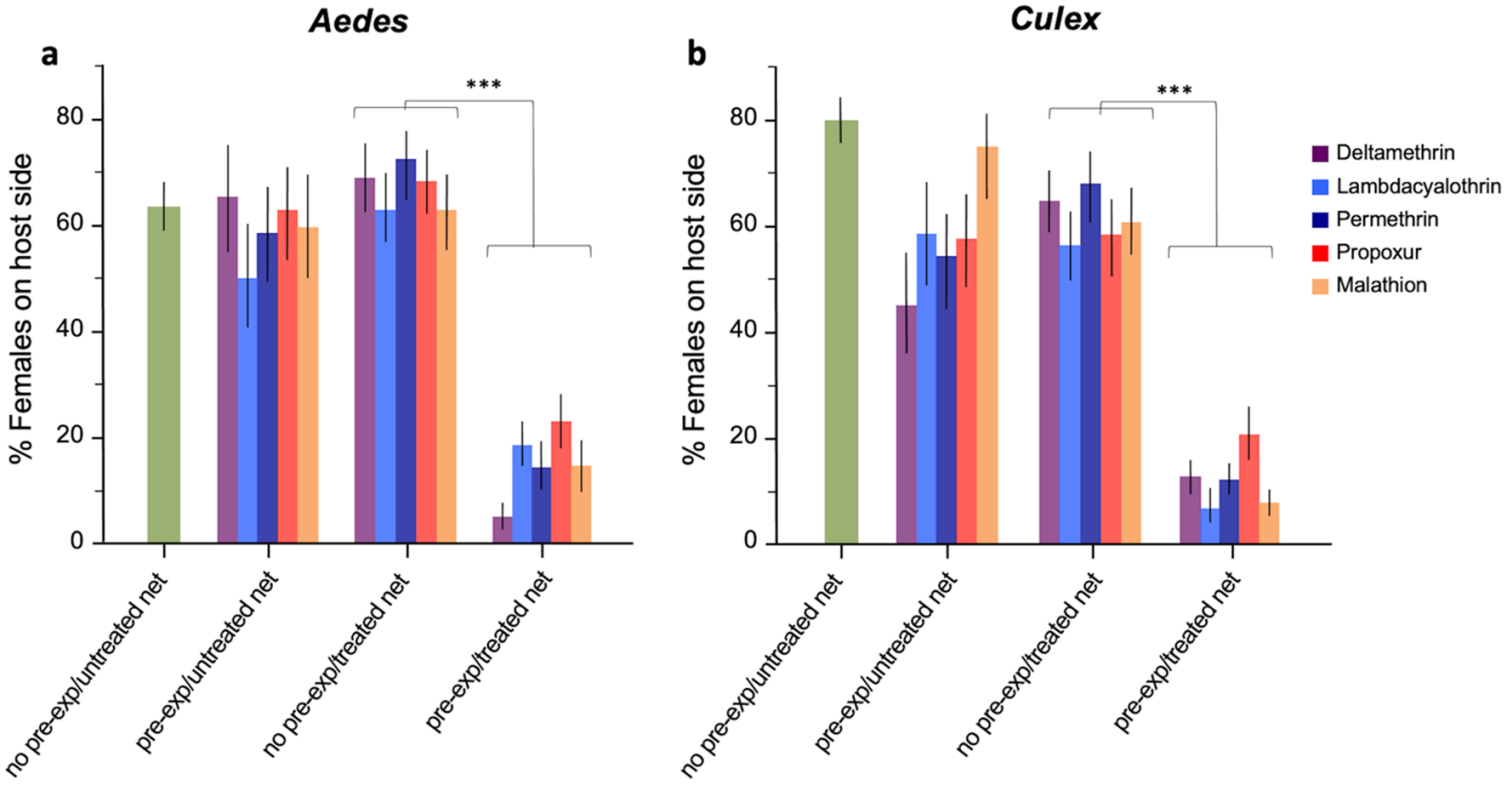
The proportion unconditioned and conditioned females of (**a**) *Aedes aegypti* and (**b**) *Culex quinquefasciatus* found on the host side when exposed to untreated and treated bed nets during the tunnels test. Error bars represent 95% confidence intervals. *P* values are *P* < 0.001 ***.

When faced to untreated nets, over 56.2% of unconditioned and 59% (range 50.5–65.4%) of conditioned female *Ae. aegypti* were found in the host side (Fig. 2a), reflecting the high feeding rate found in these both groups. The same groups exhibited different behaviour when exposed to pesticide nets. Strong avoidance behaviour was noted in the conditioned group with a low proportion of females found in the host side (mean = 25.0%, range 14.9–52.1%) compared to the naïve group (mean = 67.5, range 63.6–69.1%). The difference between both groups was significant for all pesticides tested (Likelihood Odds ratio: *P* < 0.001 in all cases, Table 1).

In *Cx. quinquefasciastus*, most conditioned females avoided contact with the pesticide nets and were found in the main compartment away from the host. Only 12.1% of females (range 6.9–21.1) were found in the host compartment, which was 5-times less than the proportion of non-pre-exposed females (mean 61.9%, range 56.9–67.9%) (Likelihood Odds ratio: *P* < 0.001 in all cases) (Fig. 2b).

Comparison of non-pre-exposed females with a treated versus a non-treated net revealed significant repellency against all pesticides for *Culex* (Likelihood Odds ratio: LR > 1.68, *P* < 0.012 in all compounds). In *Aedes*, there was no repellent effect in any of the compounds (Likelihood Odds ratio: *P* > 0.05 for all compounds).

#### Female survival

**Figure 3:**
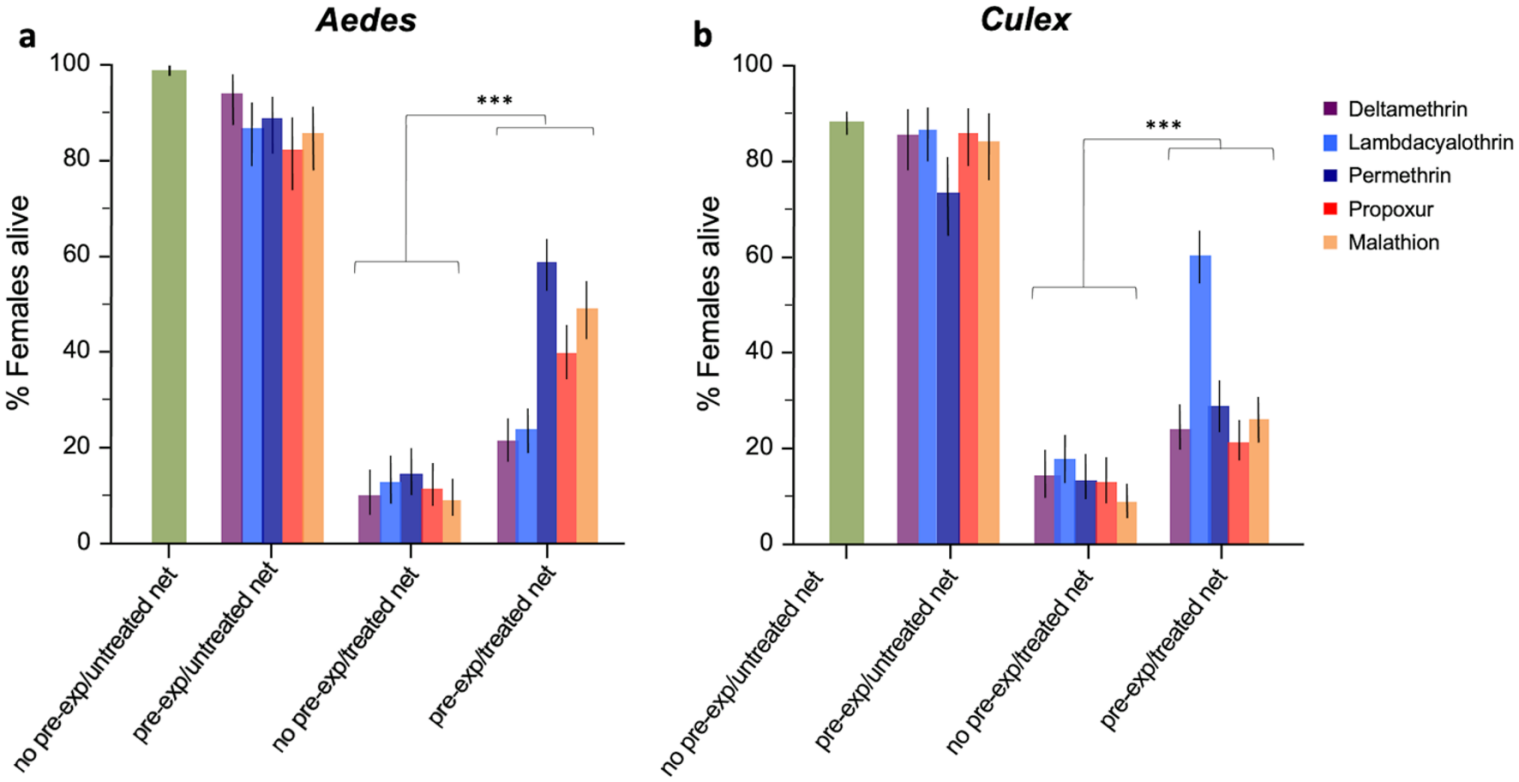
Proportion of surviving unconditioned and conditioned (**a**) *Aedes aegypti* and (**b**) *Culex quinquefasciatus* females exposed to untreated and treated bed nets in the WHO tunnel tests. Error bars represent 95% confidence intervals. *P* values are *P* < 0.001 ***.

Faced with untreated control nets, unconditioned and conditioned female *Ae. aegypti* were able to fly across the tunnel and survive with a mean survival rate of 98.9% and 87.5% (range 82.3–94.1) respectively (Fig. 3a). The survival rates in these two control groups were drastically higher compared to the survival rate of the unconditioned group that crossed treated nets over to the host compartment of the tunnel (mean 11.5% range 8.9–14.4%). However, the conditioned females, pre-exposed to pesticide compounds, avoided the treated nets and forewent blood feeding, thus displaying a survival rate threefold higher (mean 38.34% range 21.2–58.4%) than the unconditioned females exposed to pesticide nets (Likelihood Odds ratio: *P* < 0.001 in all compounds) (Table 1, Fig. 3a).

Similar outcomes were reported for *Cx. quinquefasciastus* where survival of non-pre-exposed and the pre-exposed females control groups in presence of an untreated net, resulted in a mean survival of 88.8% and 83.3% (range 73.8–86.3), respectively (Fig. 3b). However, in the presence of pesticide treated nets, non-pre-exposed *Cx. quinquefasciatus* females incurred very high mortality (mean survival 12.9%, range 8.0–17.6) whilst pre-exposed females avoided crossing the net, resulting in significantly higher survival (mean 32.1%) (Likelihood Odds ratio: *P* < 0.0001 in all cases) (Table 1 and Fig. 3b). The difference in survival between these two groups was the highest for Lambdacyhalothrin 0.05% (60%) and lowest for Propoxur 0.1% (21.1%) (Fig. 3b).

### Tests of associative learning - Resting site choice test

**Figure 4:**
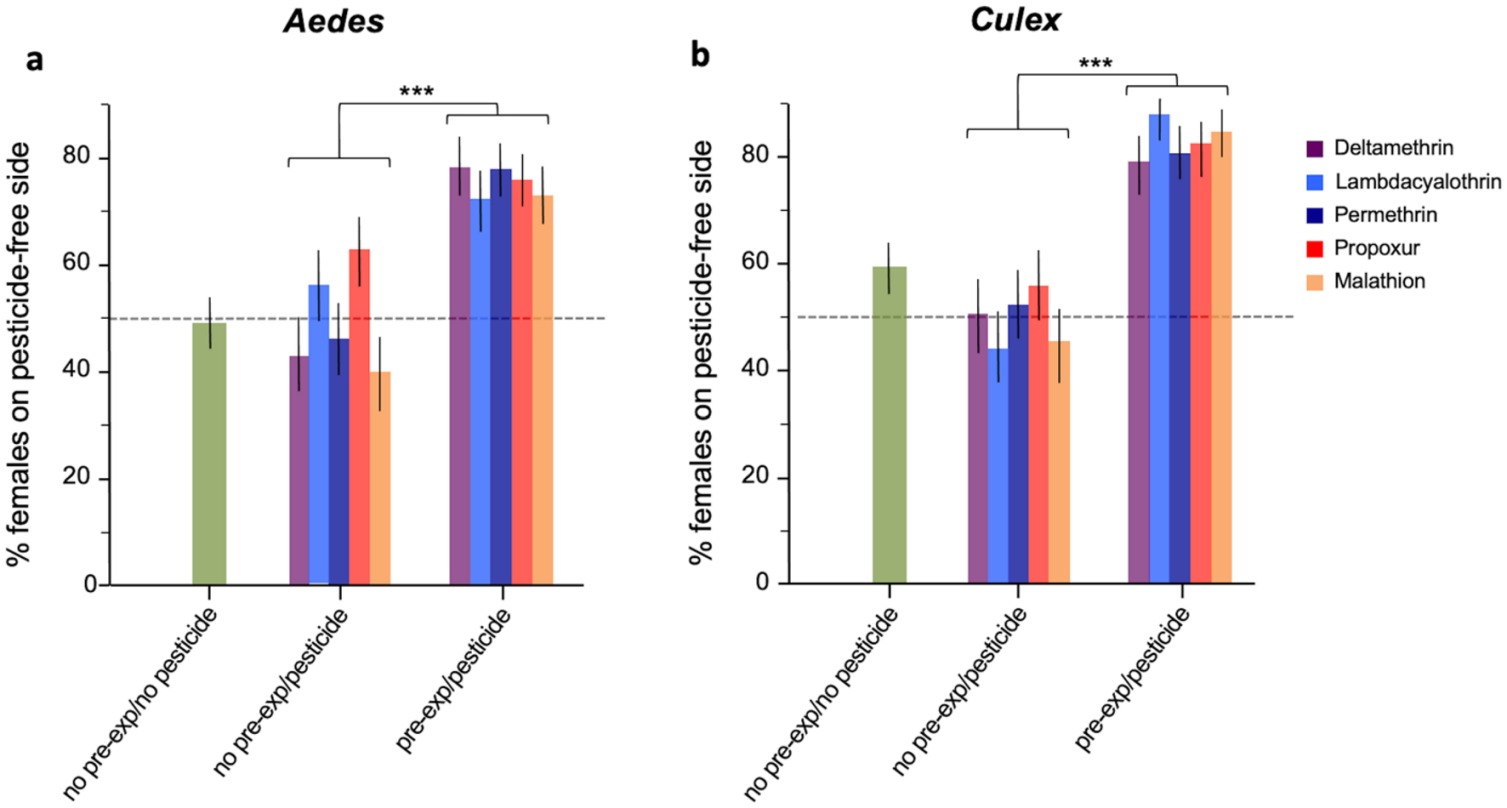
Proportion unconditioned and conditioned females of (**a**) *Aedes aegypti* and (**b**) *Culex quinquefasciatus* resting in the pesticide free side when faced to the choice compartments with pesticide smell and no pesticide smell. Error bars represent 95% confidence intervals. *P* values are *P* < 0.001 ***.

Unconditioned *Ae. aegypti* females faced with a choice of resting site compartments with no pesticide smell, did not exhibit any inherent preference between the left (49.41%) or right (50.59%) compartments (Chi-square Goodness of fit: *P* > 0.05). Likewise, no significant avoidance was reported in the naïve group faced to a choice of pesticide or control. The mean proportion of females resting in the left compartment was 49.78% (range 42.10–63.16) and 50.22% (range 36.8–59.9) in the right one (Chi-square Goodness of fit: *P* > 0.05 in all cases) (Fig. 4a). However, conditioned *Ae. aegypti* females displayed a strong significant avoidance of the compartment with the pesticide tube. In this group, on average 75.69% of females (range 72.4–78.3) preferred to rest in the compartment with the control tube. The difference in the proportion of females choosing the pesticide-free side between the conditioned and unconditioned females groups was highly significant for all insecticides tested (Likelihood Odds ratio: *P* < 0.001 in all cases, Table 2).

**Table 2:** Logistic regressions testing the effects of the 3 treatment groups and replicates on the proportion of females that rested in the compartment void of pesticide smell during the resting site choice assay.

Dependent variables	Species	Factors	Deltamethrin		Lambdacyalothrin		Permethrin		Propoxur		Malathion	
			LR^†^	*P* value	LR	*P* value	LR	*P* value	LR	*P* value	LR	*P* value
Pesticide-free compartment %	*Ae. aegypti*	Treatment	73.7	< 0.001	34.01	< 0.001	67.1	< 0.001	51.3	< 0.001	58.1	< 0.001
		Replicate^§^	7.5	0.379	8.9	0.263	6.9	0.436	4.9	0.677	10.3	0.173
	*Cx. quinquefasciatus*	Treatment	50.5	< 0.001	99.7	< 0.001	44.7	< 0.001	42.3	< 0.001	94.2	< 0.001
		Replicate	46.4	< 0.001	41.2	< 0.001	44.3	< 0.001	38.1	< 0.001	33.6	< 0.001

All dependent variables were treated as binomial variable. The Chi-square of Likelihood Ratio effect tests (LR) are presented with respective *P* values.

^§^Replicate effects were nested within treatment.

Similar results were found in *Cx. quinquefasciatus,* although there was a small but significant departure from 50:50 in the control group with no pesticide smell in terms of the compartment unconditioned females preferred to rest in. On average 40.56% of females rested in the right compartment and 59.44% in the left. In naive non-pre-exposed females, there were no marked preference for resting in the compartment with a pesticide tube and the pesticide free side. A mean value of 50.38% (range 43.63–55.49) of females was found on the pesticide-free side and 49.21% range (44.55–56.37%) on the pesticide side (Fig. 4b). In contrast, conditioned pre-exposed females strongly preferred resting on the pesticide free side (mean 83.1% range 79.3–87.9) and this differed significantly from any of the control groups (Likelihood Odds ratio: *P* < 0.001 in all cases, Table 2).

## Discussion

This study provides the first comprehensive experimental evidence of learned pesticide avoidance in females of two major vector species, *Ae. aegypti* and *Cx. quinquefasciatus*. Associative learning was achieved through exposure to sub-lethal doses of deltamethrin, permethrin, lambdacyalothrin, propoxur and malathion, 5 of the chemical compounds most used for vector control and belonging to the three major chemical classes, pyrethroids, carbamates and organophosphates. As a result of a single sub-lethal exposure to pesticide females of both species avoided passing through the pierced treated bed net of the WHO tunnel assays to feed on the host. These females forewent blood-feeding for over 12 h and many died unfed. Furthermore, in resting site choice assays, pre-exposed females displayed a strong preference for a resting site away from the smell of pesticides. In contrast, naive unconditioned females facing the same two assays were decimated by pesticide exposure in the tunnel assay. In the resting site choice test assay, unconditioned females were not significantly repelled by the smell of pesticide compounds and used each compartment indiscriminately.

The strong learned avoidance responses of *Ae. aegypti* and *Cx. quinquefasciatus* were consistent across both the tunnel and resting assays for all pesticides used during conditioning. This may not be surprising given the attention paid to conditioning females with amounts of each pesticide with comparable sub-lethal effect (30–40% mortality). This effectively amounted to a reinforcing stimulus of comparable strength across all compounds, replicates and experiments. The results also suggest that all pesticides provided olfactory stimuli of sufficient intensity so that females could associate with their toxic effects.

Taken together, these findings confirm that pesticide avoidance learning may be a simple ubiquitous mechanism enabling mosquitoes to maximize survival in environments becoming increasingly challenging due to intensification of chemical control interventions carried out in countries endemic for vector-borne diseases^38–43^. Unlike metabolic, target site or cuticular pesticide resistance mechanisms, pesticide avoidance learning does not prevent naive mosquitoes from being killed by pesticides. Importantly, when a first exposure is not lethal, surviving females may subsequently detect the smell of pesticides and systematically avoid them to seek safer blood feeding and resting site opportunities, thus surviving to reproduce. It is noteworthy that sub-lethal exposures may be an extremely common situation for mosquitoes, particularly in vector populations that already possess multiple physiological pesticide resistance mechanisms. Pesticide sub-lethality is also inherent to various scenarios of bed net aging, deterioration and missuses which are compounded by infrequent replacement^44–47^. The same applies to IRS and space spraying interventions which may again target resistant mosquito populations or be conducted at intervals that create many opportunities for sub-lethal exposures^48,49^.

The extent to which and how associative learning contributes to pesticide behavioural avoidance (deterrence) and its interplay with contact irritancy and non-contact repellency remains an open question. Behavioural resistance components are not often detailed as part of vector control^29,50^. These tend to prioritize tube and cone assays, two methods that force mosquitoes into prolonged direct contact with pesticides^36,37^. The tunnel assay is the recommended assay for evaluating impregnated bed net deterrence^36^. However, it is a more complex assay that relies on animal use, and therefore cannot be conducted as frequently. In this study, the majority of conditioned, pre-exposed females were recovered in the main compartment, away from the host compartment -suggesting that they stayed away from the harmful contact of the treated net. The resting site choice assay was designed so that mosquitoes would not experience direct contact with pesticides and thus could neither exhibit contact irritancy nor learn from the negative effects experienced during the 16 h-long assay. The results of both assays suggest that pre-exposed females displayed spatial non-contact repellency when detecting pesticide smell. We also found some evidence of low levels of innate repellency in *Cx. quinquefasciatus* but not in *Ae. aegypti*.

There has been a recent technological push to develop tools enabling a better understanding of mosquito behaviour as they approach bed nets and other vector control interventions^51–54^. Studies of associative learning could greatly benefit from these advances. Some of the recently-developed 3-dimensional imaging systems^51–54^, combined with semi-field experiments would provide ideal platforms for disentangling processes of learned avoidance from those of innate contact irritancy and spatial repellency. Importantly, the results of this study suggest that behavioural experiment designs should take into account associative learning a-priori, which might otherwise be undetected or misinterpreted, particularly when experiments focus on recently-emerged lab-reared naive mosquitoes.

Further studies will also be needed to assess the extent to which pesticide avoidance learning contributes to the behavioural plasticity and behavioural changes observed in mosquito communities facing intense chemical control selection pressures^50,55^.

*Aedes aegypti* and *Culex quinquefasciatus* females are able to learn to avoid the smell of and their exposure to insecticides in order to maximize their survival. Thereby reducing the efficacy of current chemical vector control intervention and confirming the importance of associative learning in behavioural resistance. Associative learning offers a new paradigm for re-evaluating complex patterns of repellency, pesticide deterrence and mosquito phenotypic plastic and evolutionary responses to pesticides that affect the impact of chemical control interventions beyond simple genetic resistance mechanisms. Ultimately, a better understanding of mosquito cognition and nociception may be key to giving a second wind to chemical control interventions, improving integrated pest management programs or designing novel vector control approaches that elude mosquito behavioural resistance.

## Methods

### Mosquito rearing

All experiments were conducted with the long-established VCRU reference susceptible strains of *Ae. aegypti* and *Cx. quinquefasciatus* in the laboratories of the Vector Control Research Unit (VCRU) at the University Science Malaysia (USM). The VCRU is a WHO-collaborating Centre responsible for regional testing of chemical mosquito control tools and the global distribution, preparation, storage and improvement of WHO test kits for pesticide resistance assays. The mosquito learning experiments made use of well-established WHO assays and were conducted under ethical permissions from the USM ethics committee (ethical approvals number: 390 and 645). Both strains were reared under standard VCRU insectary conditions, at 27 ± 2 °C and 80 ± 10% relative humidity under subdued light. The strains were maintained on mice blood by inserting trapped mice inside the rearing cage. Two days following blood-feeding, *Cx. quinquefasciatus* laid eggs on the water and *Ae. aegypti* on filter paper. The eggs were then transferred to several petri dishes that contained season water to hatch. Once hatched, the larvae were fed daily with two teaspoons of powdered food containing a mixture of cat biscuits, milk powder, yeast, beef, and liver. The emerging pupae were pipetted out individually and transferred into a beaker before placing them into the rearing cages. All the rearing cages used in this study were of the same size and shape (cube shaped with a size of 25 × 25 × 25 cm) and covered with polyester net.

### Mosquito conditioning using tube tests

Conditioning between the smell of pesticide compound (olfactory conditioning stimulus) and the negative effect of pesticides on mosquitoes (reinforcing stimulus) was performed using the well-known pesticide bioassay tube test devise^37^. The standard assay was first used to confirm that both reference susceptible strains were still fully susceptible to every compound used when following the standard WHO procedures with standard pesticide-impregnated exposure papers^37^.

**Figure 5:**
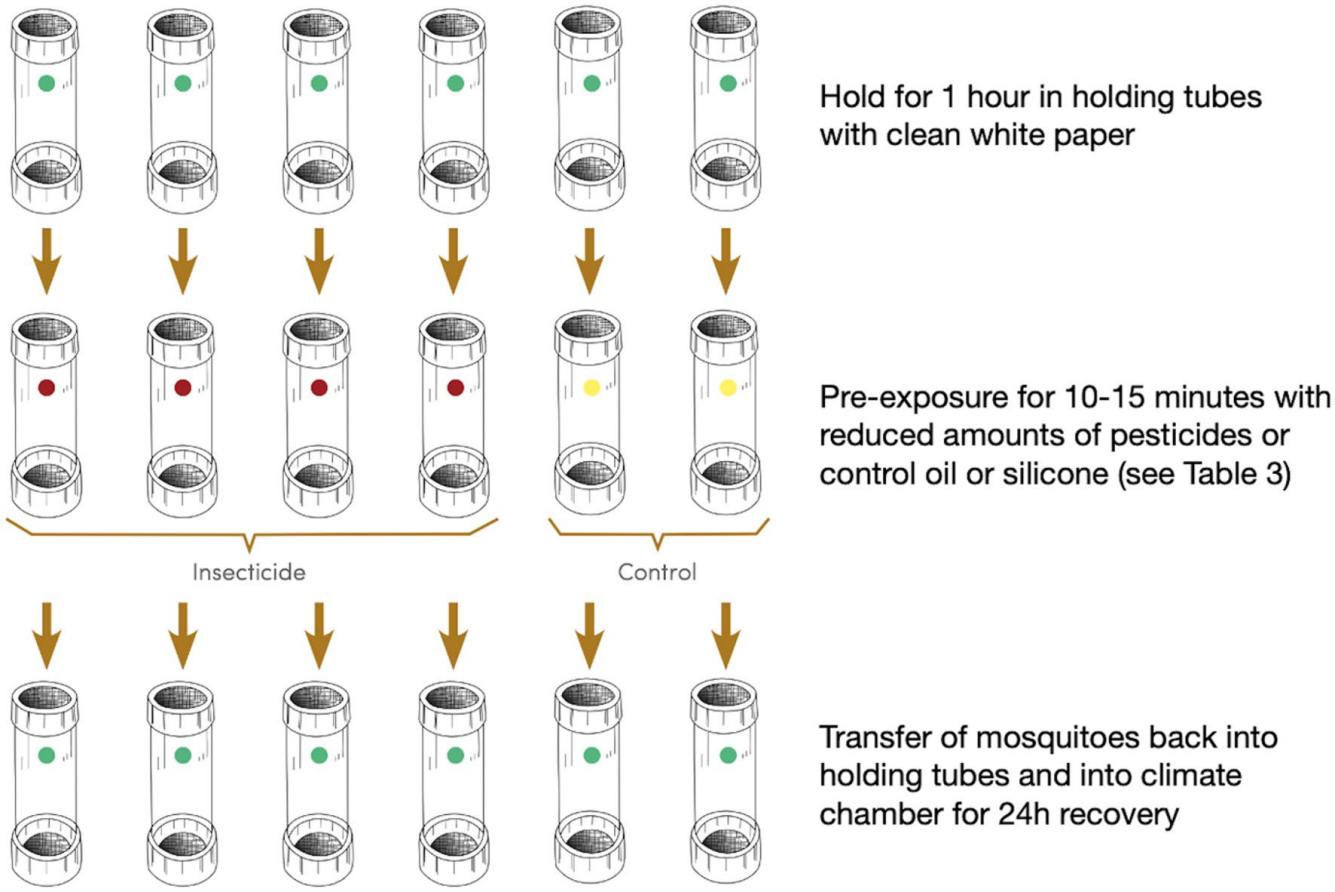
Mosquito conditioning using WHO's pesticide bioassay tube test system (figure adapted from (WHO 2016)). The assay was modified so as to subject the majority of mosquitoes to a sub-lethal dose of pesticide to induce conditioning through association of the pesticide smell with toxic-effects. Pre-exposed conditioned female mosquitoes were then subjected to 2 assays of associative memory (see Methods and Fig. 6a, b).

Next, two groups of female mosquitoes were used for conditioning using the same tube assay devices, but with modified procedures (Fig. 5). A first group of females were pre-exposed (pre-exp) and conditioned to associate the smell of insecticides with the detrimental effects associated with their toxicity. Under normal circumstances, the toxic effects of pesticides on susceptible mosquitoes produce mosquito knockdown followed by death^28,56,57^. In this study, a method was devised to expose the majority of females to sub-lethal doses of pesticides so that the majority of individuals were negatively affected or even knocked-down, but had fully recovered 24 h after exposure (see details below). The 2nd group was the no pre-exposed group (no pre-exp). This control group was exposed with the smell of silicon oil or olive oil used in the formulation of each pesticide used for the pre-exp group. In WHO insecticide susceptibility tube tests, silicone oil is used as control paper for pyrethroid compounds and olive oil for carbamates and organophosphates^37^. Silicon oil or olive oil do not cause any negative effects on mosquito health. Therefore, in the mosquito conditioning stage of this experiment, the smell of the oils could not be associated with any negative stimulus.

In each of tube test, 20 randomly picked three-to-six day old female *Ae. aegypti* and *Cx. quinquefasciatus,* that had not yet blood-fed, were aspirated out from the rearing cages and gently blown into yellow-dotted transfer tubes that were lined inside with normal papers. Pre-exposed individuals were transferred into pre-exposure tubes labelled with a red sticker and lined with normal paper on which pesticide was transferred by rubbing firmly for 5 min with paper impregnated with insecticides. Three different classes of insecticides including the organophosphate (malathion 5%), the carbamate (propoxur 0.1%) and the pyrethroid (Deltamethrin 0.05%, Permethrin 0.75% and Lambda-cyhalothrin 0.05%) were used throughout all the experimental tests in this study. Transferring a smaller amount of pesticide onto the fresh paper was necessary to reduce the lethal dose to a sub-lethal dose for the susceptible reference strains. The optimal total time for the rubbing process and exposure to insecticide varied for each compound. The optimal times were determined from a large number of iterative preliminary optimization experiments which generated mortality curves whereby the insecticide dose applied and exposure time to insecticide smell was enough to ultimately kill approximately 30% of female, leaving the 70% remaining in the “knocked-out” state but not dead (after 24 h recovery) (Table 3). Mosquitoes were kept in the pre-exposure tubes for 10 to 15 min according the species and the pesticide (Table 3) before being transferred placed back into the transfer tubes of the tube assay device (Fig. 5). Thirty percent mortality rate was an important target to achieve to ensure that surviving mosquitoes conditioned with the smell of insecticides did not simply constitute as small fraction of an unknown, particularly resistant, phenotype. After completion of the pre-exposure conditioning step, cotton wool soaked with 10% sucrose solution was placed on top of the mesh of the transfer tubes before putting them into the climate chamber for 24 h of recovery (5 pm–5 pm next day).

**Table 3:** Optimised pesticide concentration and duration of transfer used in preparing for sub-lethal mosquito conditioning exposure using WHO tube tests (see Methods for details).

Species	Pesticide concentration (%)	Control paper	Transfer duration (min)	Exposure time (min)
*Aedes aegypti*	Deltamethrin 0.05	Silicon oil	5	15
	Permethrin 0.75		5	15
	Lambda-cyhalothrin 0.05		5	15
	Propoxur 0.1	Olive oil	5	15
	Malathion 5		5	10
*Culex quinquefasciatus*	Deltamethrin 0.05	Silicon oil	5	15
	Permethrin 0.75		5	15
	Lambda-cyhalothrin 0.05		5	15
	Propoxur 0.1	Olive oil	5	15
	Malathion 5		5	15

The same procedure applied for individuals of the non-pre-exposed group that were transferred into green-dotted tubes indicating control tubes. However, the inner surface of the control tubes was lined with normal paper that was rubbed with control papers. Two types of control papers used in this test were PY-Control (paper impregnated with the smell of silicone oil) and OP-Carbamate control (paper impregnated with the smell of olive oil). The PY- control and the OP-Carbamate control were used to replace the paper impregnated with insecticides belonging respectively to the pyrethroid group and the carbamate-organophosphate group. This group followed the same recovery pattern as the group pre-exposed to insecticide smell.

As per WHO guidelines, papers that contained a sub-lethal insecticide dose and/or a reduced dose of PY-control and OP-Carbamate control were reused only for two replicates before changing to a new one.

A total of 100 mosquitoes were needed for each insecticide. However, due to the expected 30% mortality, a total of 200 mosquitoes equivalent to 10 tube tests for each insecticide, were used—this constituted one replicate in the pre-exposed group. The test with the control group was performed with 100 mosquitoes equivalent to 5 tube tests for one replicate as exposure to both odours did not cause mortality.

### Tests of associative learning - WHO tunnel assay

**Figure 6:**
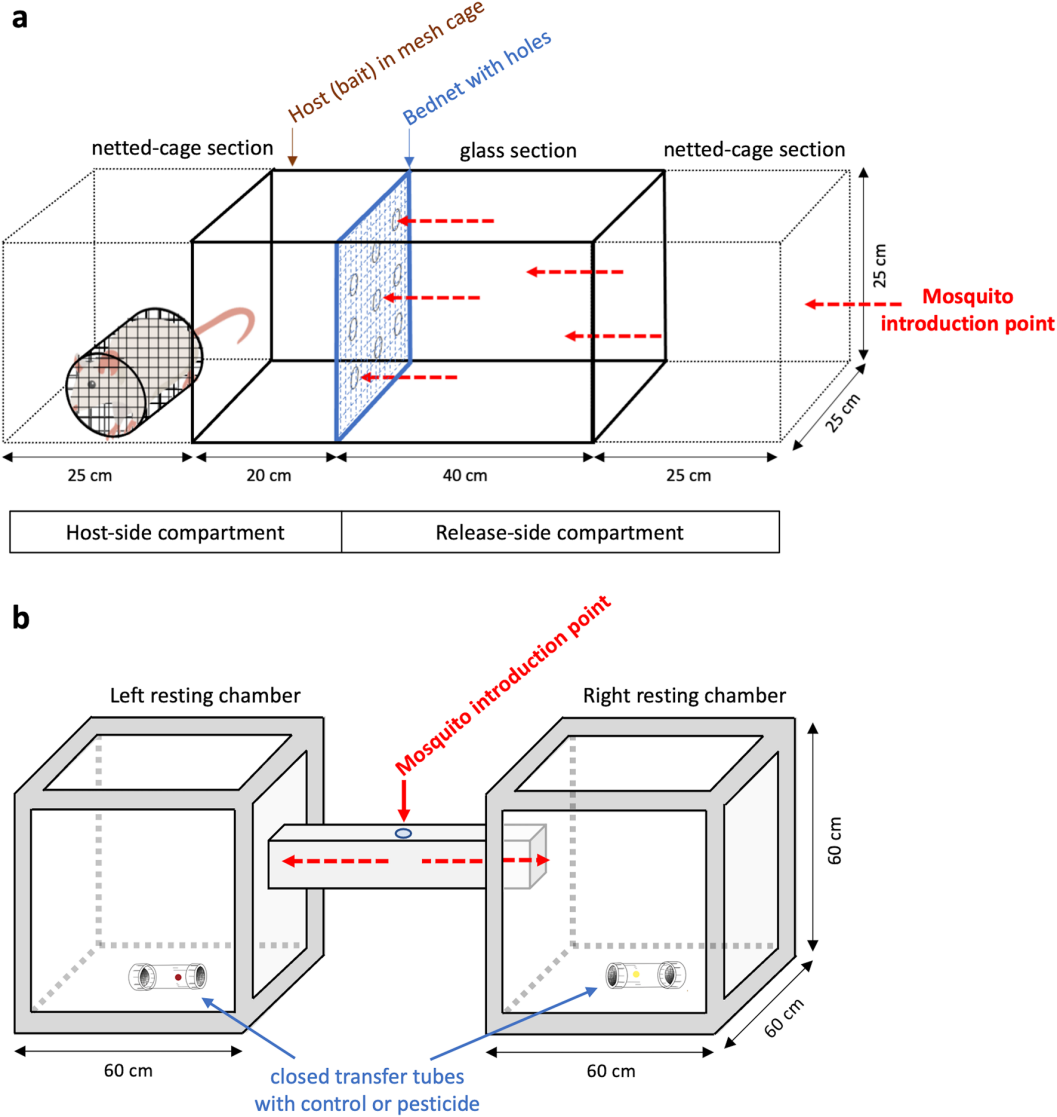
Experimental testing of associative learning**.** (**a**) The WHO Tunnel Tests measures the deterrence and killing efficacy of bed nets (WHO 2013). (**b**) The Resting site choice tests uses 2 aluminium framed Perspex compartments linked by a bridge to measure the level of repellency.

The WHO tunnel assay was used to test whether pre-exposed (pre-exp) females had learned to associate pesticide smell with their toxic effects compared to non-pre-exposed females (no pre-exp). This test is widely used to assess the excito-repellency of bed nets treated with various formulations (WHO 2013)^36^. This test was conducted using a square tunnel made of glass panels siliconed together with a size of 60 × 25 × 25 cm (L × W × H) following WHO guidelines (Fig. 6a). Two cubic metal-frame cages of dimensions 25 × 25 × 25 cm (L × W × H) covered with a polyester net sleeve. Each netted cage section was connected to either end of the main glass section to create a tunnel with left and right extensions. Two wooden frames of the same height and width as the tunnel ensured that the net sleeves of the cages were perfectly secure and connected to the main glass section. The left netted cage of the tunnel contained a white rat which acted as bait. The glass section of the tunnel was separated into a smaller left (L 20 cm) and larger right section (L 40 cm) by a piece of bed net attached to a clipboard with nine holes of the same size (Fig. 6a). Each hole has a diameter of 1 cm and equidistant of 5 cm from each other. Treated or untreated squares of bed net fabric were used to separate the larger from the smaller compartment (see experimental and control groups below). When treated net was used, it was rubbed with paper impregnated with the same insecticides used for conditioning via pre-exposure. The untreated net was rubbed with silicone or olive-oil impregnated control papers similar to ones used for the control group, that was not pre-exposed to insecticide smell (PY-Control and OP-Carbamate). The insecticide-impregnated papers and control paper were rubbed vigorously on the nets for 5 min (in the same way as the tube test). The experiment was conducted under a controlled temperature of 26 ± 2 °C and 60–80% of humidity.

During the test, 100 females from the two conditioning stage groups (pre-exp or no pre-exp females) were released into the right compartment through a sleeve of the netted cage at 5 pm, directly after the 24 h recovery period from the conditioning step was completed. They were left in the tunnel until 9 am the following day. The net used to separate the larger from the smaller compartment was replaced in every new test with a new net that had been treated with the described rubbing procedure.

Since the tunnel assays were performed with pesticide treated bed net fabric or control bed nets rubbed with oil, the design resulted in four different treatment groups with one experimental conditioned female group and three control groups:

1. The first group (pre-exposed/treated net) was the experimental group with female mosquitoes that were pre-exposed to insecticide and released into the tunnel with a net that was treated with the same insecticide. This group was the only group that was exposed to the same insecticide twice.
2. The first control group (no pre-exp/untreated net) was made of female mosquitoes that were not pre-exposed to insecticide smell and released into the tunnel with an untreated net. The untreated net was rubbed with PY-control (silicone oil) for female mosquitoes exposed to PY-control in tube tests, whereas untreated net rubbed with OP-Carbamate control (olive oil) was used for female mosquitoes pre-exposed to OP-Carbamate controls in tube tests.
3. The 2nd control group (pre-exp/untreated net) constituted female mosquitoes that were pre-exposed to insecticide smell and released into the tunnel that contained an untreated net. As in the previous group, net rubbed with PY-control was used for mosquitoes that were pre-exposed to the pyrethroid group, whereas the untreated net rubbed with OP-Carbamate control was used for mosquitoes pre-exposed to organophosphate and carbamate.
4. The 3rd control group (no pre-exp/treated net) was female mosquitoes that were not pre-exposed to insecticide smell and released into the tunnel with a net that was treated with insecticide. Females used in this test when the net was rubbed with pyrethroids were taken from the group that was exposed to PY-control, whereas net rubbed with carbamate and organophosphate group was used when females were exposed to OP-Carbamate control in tube test.

#### Data collection

At the end of the tunnel assay, the number of live and dead mosquitoes found in the left host compartment, the main glass compartment and right netted cage were counted. For all experimental groups, females were also scored as engorged (presence of blood in abdomen) or unfed (no blood present). This procedure was applied for both *Ae. aegypti* and *Cx. quinquefasciatus*.

Whether pre-exposed females had learned to associate the toxic effects of a pesticide with its smell was inferred by comparing the proportion of females that had avoided passing through the treated net to blood-feed on the bait in group 1 versus group 4. It was assumed that females pre-exposed to insecticides that subsequently avoided passing through the treated net did so in response to learning, whereas non pre-exposed females that chose to pass through the treated net did so because they did not have the opportunity to learn and memorize the smell of insecticides. Innate deterrent effects of pesticide compounds were inferred though comparisons of the proportion of mosquitoes passing through the net in group 2 versus group 4. For both species and each pesticide, group 1 was triplicated, group 2 was conducted 6 times, group 3 was conducted once, and group 4 was duplicated.

### Tests of associative learning - Resting site choice assay

Further testing of learned pesticide avoidance were performed using a resting site choice experiment specifically designed for that purpose. To do so, we used two large cubic 60 × 60 × 60 cm compartments made of aluminium frames and transparent Perspex walls inter-connected via a 60 cm Perspex bridge (Fig. 6b). The 15 × 15 cm (W × H) bridge linked the upper right side panel of one compartment to the left-side panel of the other, allowing movement of mosquitoes between the two enclosures. A petri dish with cotton soaked in sucrose solution and a plastic tube from the tube test device (described above) with its inner surface lined with paper impregnated with different pesticides or silicone control were prepared and placed in each compartment. The tube ends were closed with mesh on both ends such that female mosquitoes could not get into contact with the pesticide or control silicone. In contrast to the conditioning stage, the impregnated paper used to line the inner surface for this experiment was not rubbed onto any other paper, therefore no alteration of concentration was made from standard WHO-impregnated papers and the odours were thus stronger than in previous assays. For each test, 100 mosquitoes were released through a window located in the middle of the connecting bridge. In addition to testing pre-exposed females, two control groups were submitted to the assay resulting in 3 experimental groups:

1. The 1st group (pre-exp/pesticide) was the experimental group where female mosquitoes released on the middle bridge had been pre-exposed to insecticide smell using the tube assay conditioning step and had to choose between a compartment with pesticide smell or not. Here, the inner surface of the tube in the left compartment was lined with paper that contained an unaltered WHO concentration of insecticide (lethal dose) and the one in the right compartment was with control paper. When the inner surface of the tube in the left compartment was lined with a pyrethroid-impregnated paper, PY-control paper was used to line the inner surface of the tube in the right compartment. The females used were pre-exposed to PY-control in the previous tube conditioning step. When the inner surface of the tube in the left compartment was lined with organophosphate and carbamate impregnated paper, OP-Carbamate control paper was used in the tube in the right compartment and females were pre-exposed to OP-Carbamate control papers.
2. The first control group (no pre-exp/no pesticide) were female mosquitoes that were not pre-exposed to insecticide via tube assay conditioning had to choose between two compartments with no pesticide in the tubes. The inner surface of both tubes in each compartment was lined with the same type of control papers. Since there was no insecticide used for this group, when the inner surface of both tubes was lined with PY-control paper, the mosquitoes released were pre-exposed to PY-control, whereas when the inner surface of both tubes were lined with OP-Carbamate control, the mosquitoes released were pre-exposed to OP-Carbamate control. The controls alternated between these two types and served to highlight a potential preference for the left or right compartment independent of the effect of pesticide smell. It also served to show that cleaning and airing of the compartments effectively removed any possible remanence that could confound the next assay performed using the same enclosures.
3. The second control group (no pre-exp/insecticide) were female mosquitoes that were not pre-exposed to insecticide but had to choose between a compartment with pesticide paper or control as described for group 1. The different types of the paper lining of the inner surface of the two tubes were identical to those described for the pre-exposed female treatment group 1. When insecticide-impregnated paper was used in the tube in the left compartment, control paper was used in the tube on the right.

Female mosquitoes were released at 5 pm (right after the completion of 24 h recovery from tube test) and were left until 9 am the following day. Bioassays for group 1 and each compound were replicated 3 times, for group 2 they were performed 5 times, and for group 3 twice.

#### Data collection

At the end of each assay, the number of females (alive or dead) found in both compartments were counted. Those found in the connecting tunnel were not included in the analyses. Associative learning was measured as a significant preference for resting in the compartment away from that with the pesticide impregnated paper (pesticide-free side), whereas females in control groups were expected to be indifferent to what compartment they chose to rest in.

### Statistical analysis

Statistical analyses were carried out using the JMP 14.0 software (SAS Institute. Inc.). Logistic regressions were performed to test the effect of the treatment groups and replicates (nested within treatments) on the binomial variables: proportion of females that blood-fed, survived and found in the host section or pesticide free side in the tunnel assays; and pesticide or pesticide-free compartment in the resting side choice assays. Likelihood odds ratios from the Logistic regressions were used for *post-hoc* pairwise treatment comparisons for each pesticide tested. Chi-square tests of randomness were also used to test for departure from 50:50 in the proportion of females found in either compartment of the resting side choice assay. The lower and upper intervals 95% confident intervals (CIs) of proportions are presented in all figures for ease of comparisons between treatments and for each insecticide tested.

### Ethical considerations

All methods were carried out in accordance with relevant guidelines and regulations.
